# The role and research progress of serine metabolism in tumor cells

**DOI:** 10.3389/fonc.2025.1509662

**Published:** 2025-04-08

**Authors:** Hanning Lyu, Shuchang Bao, Lingyun Cai, Mengke Wang, Yuxin Liu, Yang Sun, Xiaoyang Hu

**Affiliations:** School of Basic Medicine, Heilongjiang University of Chinese Medicine, Harbin, Heilongjiang, China

**Keywords:** serine metabolism, cancer, one-carbon metabolism, serine catabolism, the immunosuppressive microenvironment

## Abstract

Serine is crucial for tumor initiation, progression, and adaptive immunity. Metabolic pathways for serine synthesis, acquisition, and utilization in tumors and tumor-associated cells are influenced by various physiological factors and the tumor microenvironment, leading to metabolic reprogramming and amplification. Excessive serine metabolism promotes abnormal macromolecule biosynthesis, mitochondrial dysfunction, and epigenetic modifications, driving malignant transformation, proliferation, metastasis, immune suppression, and drug resistance in tumor cells. Restricting dietary serine intake or reducing the expression of serine synthetic enzymes can effectively slow tumor growth and extend patient survival. Consequently, targeting serine metabolism has emerged as a novel and promising research focus in cancer research. This paper reviews serine metabolic pathways and their roles in tumor development. It summarizes the influencing factors of serine metabolism. The article explores the significance of serine synthesis and metabolizing enzymes, along with related biomarkers, in tumor diagnosis and treatment, providing new insights for developing targeted therapies that modulate serine metabolism in cancer.

## 1 Introduction

Serine, a non-essential amino acid, is vital for nucleotide and lipid metabolism, serving as a key source of one-carbon (1C) units necessary for biological growth. Within cells, serine has multifunctional roles, participating in DNA and RNA synthesis, regulating cell membrane structure and function, and supporting cell growth (1, 2). The importance of serine in cancer cell proliferation differs from that in normal cells. Compared to normal cells, most cancer cells rely on glycolysis for energy procurement (3). Due to its lower production efficiency, cancer cells increase their uptake and utilization of glucose and amino acids such as glutamine (4). In rapidly proliferating cancer cells, however, glucose and glutamine consumption is often insufficient to support biomacromolecule accumulation. In this process, serine provides most of the carbon and nitrogen units required by cancer cells, which is crucial for their survival (5). Studies have shown that both endogenous serine produced from glucose and functionally acquired exogenous serine are associated with the growth of cancer cells *in vitro* and *in vivo* and functionally promote cancer progression (6–10).

## 2 Serine metabolism

Serine is the primary source for synthesizing one-carbon (1C) units, linking amino acid metabolism to nucleotide metabolism. Thus, serine occupies a central role in cellular metabolic processes.

### 2.1 Serine as a central node in biosynthesis

Serine serves as a pivotal metabolite in various biosynthetic pathways, providing essential materials for cellular biosynthesis processes (11). Through charging tRNAs, serine participates in protein synthesis and acts as a precursor for amino acids like cysteine and glycine. It also provides head groups for the synthesis of sphingolipids and phospholipids. Notably, in the folate-mediated 1C pathway, the cleavage of serine into glycine and 1C units supports the synthesis of porphyrins, thymidylate, purines, glutathione, and S-adenosylmethionine (SAM) (12).

### 2.2 Pathways for serine acquisition

Serine, essential for cell growth, is acquired through internal synthesis and external uptake.

#### 2.2.1 Internal synthesis

One-carbon metabolism includes a bicyclic pathway formed by the coupling of the folate cycle, methionine cycle, and trans-sulfuration pathway (13, 14). The folate and methionine cycles are crucially interlinked pathways in 1C metabolism, providing methyl groups for DNA, amino acid, creatine, polyamine, and phospholipid synthesis (15). One-carbon metabolism generates various products by cycling 1C units from different amino acids and integrating multiple cellular nutritional states (13). The primary sources of 1C units are the catabolic pathways of serine, glycine, and histidine. Serine and glycine are interconvertible, with serine serving as the principal donor of 1C units when converted to glycine (16). Serine is derived from the metabolism of nutrients, including proteins and phospholipids. Glycine, synthesized endogenously from serine, acquires a hydroxymethyl group through serine hydroxymethyltransferase (SHMT). Although alternative sources for serine synthesis exist, the Serine Synthesis Pathway (SSP) remains the primary supplier of serine in the body, as other sources are insufficient to meet tumor cell demands (2). The SSP is recognized as a critical growth and stress resistance pathway in cancer cells (17–21).

**Figure 1:**
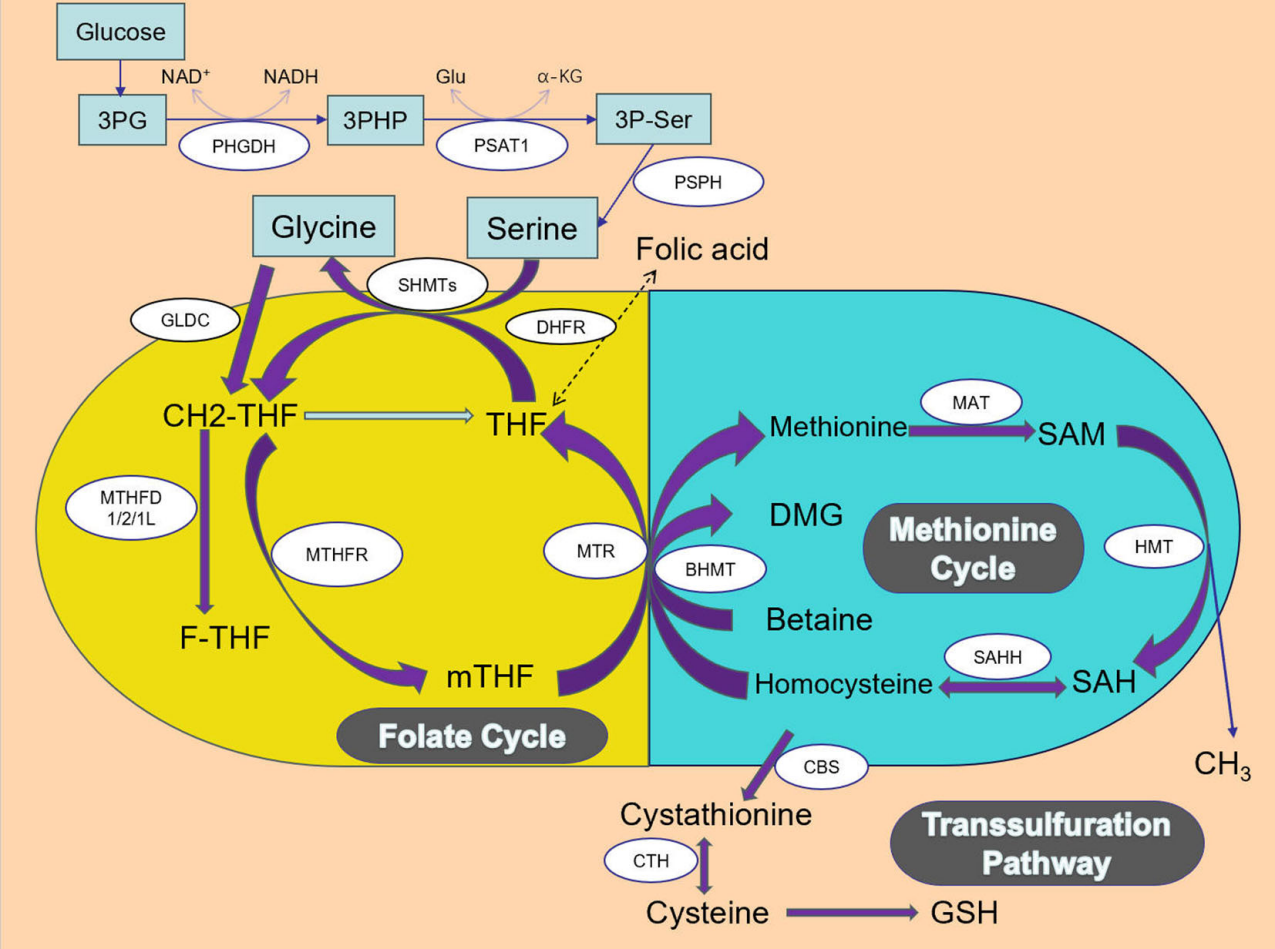
Title: One-carbon cycle.

As illustrated in **Figure 1**, in the folate cycle, folate is reduced twice by dihydrofolate reductase (DHFR), ultimately converting into tetrahydrofolate (THF). Serine is synthesized from the glycolytic intermediate 3-phosphoglyceric acid (3PG) through three steps. First, 3PG is oxidized to 3-phosphohydroxypyruvic acid (3PHP) by phosphoglycerate dehydrogenase (PHGDH), reducing NAD+ to NADH. First, 3PG is oxidized to 3-phosphohydroxypyruvic acid (3PHP) by phosphoglycerate dehydrogenase (PHGDH), reducing NAD+ to NADH. Secondly, through a transamination reaction, 3PHP receives an amino group from glutamate, producing 3-phosphoserine (3P-Ser) and α-ketoglutarate (α-KG), a step catalyzed by phosphoserine transaminase 1 (PSAT1). Finally, 3P-Ser is dephosphorylated by phosphoserine phosphatase (PSPH) to produce serine (12). Under the catalysis of SHMTs, serine is converted to glycine, and during this process, THF accepts the one-carbon unit from serine, forming 5,10-methylenetetrahydrofolate (5,10-CH2-THF). 5,10-CH2-THF can be converted to 10-formyltetrahydrofolate (F-THF) by methylenetetrahydrofolate dehydrogenase (MTHFD) 1/2/1L, or reduced to 5-methyltetrahydrofolate (mTHF) by methylenetetrahydrofolate reductase (MTHFR). mTHF can be demethylated and converted back to THF, completing the folate cycle and initiating the methionine cycle.

In the methionine cycle, mTHF transfers the one-carbon unit to homocysteine to form methionine, which is then converted by methionine synthase (MTR). Methionine is converted into S-adenosylmethionine (SAM) by methionine adenosyltransferase (MAT). SAM, a substrate for methylation reactions, forms S-adenosylhomocysteine (SAH) upon demethylation, which is converted back to homocysteine by S-adenosylhomocysteine hydrolase (SAHH), completing the methionine cycle (14). In the trans-sulfuration pathway, homocysteine is converted to cystathionine by cystathionine beta-synthase (CBS) and further to cysteine by cystathionine gamma-lyase (CTH), ultimately leading to glutathione (GSH) synthesis.

#### 2.2.2 External uptake

While some cancer cells meet their growth requirements through *de novo* serine synthesis, others depend on exogenous serine (8, 9, 22). In normal cells, serine is synthesized via the *de novo* pathway, involving reactions catalyzed by phosphoglycerate dehydrogenase (PHGDH). However, certain cancer cells may have mutations or abnormal expressions in key enzymes of these metabolic pathways, which restrict their ability to synthesize serine *de novo*. Consequently, these cancer cells often rely on exogenous serine to support their growth and metabolic needs, as observed in Ewing sarcoma and liposarcoma cells (23). The supply of exogenous serine can be achieved through the intake of a serine-rich diet or the availability of serine in the extracellular environment, where serine transport proteins play a critical role. The serine transporters encompass members of the SLC1A, SLC6A, SLC7, SLC36, and SLC38 families, with their specific classifications and functional characteristics detailed in the **Table 1**. The Solute Carrier Family 1A (SLC1A) includes two major transport systems: Alanine-Serine-Cysteine Transporters 1-2 (ASCT1-2) and human glutamate transporters (Excitatory Amino Acid Transporters 1-5, EAAT1-5). ASCTs are widely expressed and are one of the four primary neutral amino acid transport systems in the human body (24, 25). In particular, ASCT2(SLC1A5) functions as a neutral amino acid exchanger, transporting serine and other amino acids (26). Under serine-limited conditions, ASCT2 is essential for purine nucleotide biosynthesis. Combined depletion of ASCT2 with a serine-free diet induces tumor regression (27). Similarly, in activated T cells, most intracellular serine is obtained from the extracellular environment (28). The main transporter for serine uptake in T cells has not yet been identified, but SLC1A5 is hypothesized to be involved (29). Beyond ASCT2, emerging evidence has revealed ASCT1 (SLC1A4) as a functional serine transporter in astrocytes (30). In parallel, Maddocks and colleagues have identified SLC6A14 and SLC12A4 as contributors to serine transport in cancer cells (31).

This dependence on exogenous serine has significant clinical implications, providing a potential therapeutic target within the serine metabolic pathway. Interfering with cancer cells’ dependency on exogenous serine, such as by inhibiting serine transport proteins or disrupting serine uptake, may hinder cancer cell growth and proliferation. It is important to acknowledge that serine metabolism may differ among various types of cancer, and the specific dependencies and mechanisms of serine metabolism are still under investigation. A deeper understanding of cancer cell dependency on serine is crucial for developing effective treatment strategies for tumors.

**Table 1:** Classification and nomenclature of serine transporters.

Serine Transporters Associated with Tumors			
Transporter	Systematic Name	Alias/Common Name	Functional Description
ASCT2	SLC1A5	–	Primarily transports serine, glutamine, etc. Highly expressed in tumor cells, supporting tumor growth and metabolism.
SLC6A14	SLC6A14	–	Broad-spectrum amino acid transporter mediating serine and neutral amino acids. Highly expressed in various cancers.
LAT1	SLC7A5	–	Transports large neutral amino acids (including serine) via heterodimeric form. Overexpressed in tumor cells.
PAT1	SLC36A1	–	Transports small neutral amino acids (e.g., serine, glycine). May participate in metabolic regulation in tumor cells.
SNAT1	SLC38A1	–	Mediates neutral amino acids (e.g., serine, glutamine). Potentially supports amino acid uptake in tumor cells.
SNAT2	SLC38A2	–	Transports neutral amino acids (e.g., serine, alanine). May contribute to metabolic adaptation in tumor cells.
Serine Transporters Unrelated to Tumors			
Transporter	Systematic Name	Alias/Common Name	Functional Description
ASCT1	SLC1A4	–	Transports neutral amino acids (e.g., serine, alanine). Predominantly expressed in the nervous system and kidneys.
GlyT1	SLC6A9	–	Primarily mediates glycine transport but may also participate in serine transport. Plays critical roles in the nervous system.
LAT2	SLC7A8	–	Transports large neutral amino acids (including serine). Widely expressed in normal tissues.
EAAT1	SLC1A3	GLAST	Mainly transports glutamate but may also mediate serine transport. Expressed in the nervous system.
EAAT2	SLC1A2	GLT-1	Primarily mediates glutamate transport with potential serine transport activity. Highly expressed in the central nervous system.
EAAT3	SLC1A1	EAAC1	Primarily transports glutamate but may contribute to serine transport. Localized in neurons and kidneys.

#### 2.2.3 Metabolic interplay and plasticity between *de novo* synthesis and exogenous uptake

Intracellular synthesis and extracellular uptake constitute two primary pathways for serine acquisition in cancer cells, operating independently yet synergistically to sustain tumor metabolism. The *de novo* synthesis pathway (e.g., serine synthesis pathway, SSP) confers metabolic autonomy by enabling endogenous serine production, particularly under nutrient-replete conditions where SSP suffices to meet proliferative demands (21, 32). For instance, breast cancer cells with PHGDH amplification exhibit marked dependency on SSP (33). Conversely, cancers harboring mutations or dysregulated expression of SSP enzymes (e.g., PHGDH) display compromised biosynthetic capacity, leading to heightened reliance on exogenous serine uptake. This dependency is prominently observed in Ewing sarcoma and liposarcoma (23).

Metabolic plasticity serves as a hallmark of cancer cell adaptation to microenvironmental heterogeneity. Studies demonstrate that cancer cells dynamically rewire serine acquisition strategies based on extracellular serine availability: downregulating SSP when exogenous serine is abundant, while upregulating SSP under serine-depleted conditions (10, 34–36). This bidirectional regulation not only enhances survival under nutritional fluctuations but also underpins adaptive resistance to therapeutic stress.

### 2.3 Serine catabolism

Serine catabolism serves as a central node linking cellular energy supply and biosynthetic processes. In mammals, serine degradation is predominantly catalyzed by serine hydroxymethyltransferase (SHMT), which transfers the β-carbon of serine to tetrahydrofolate (THF), generating glycine and 5,10-methylenetetrahydrofolate (5,10-CH_2_-THF). This latter metabolite functions as a one-carbon unit donor, directly participating in the synthesis of purines, thymidylate, and glutathione (11).

Serine catabolism plays pivotal roles in various physiological and pathological contexts. During respiratory impairment, it contributes to NADH regeneration (37), while in hepatic tissues, it generates NADPH to support lipogenesis (38). Under hypoxic conditions, serine catabolism regulates mitochondrial redox homeostasis. Notably, the mitochondrial isoform SHMT2 is highly expressed in proliferating cancer cells, where it enhances one-carbon metabolism to fuel nucleic acid biosynthesis and tumor progression (39). Accumulating evidence positions SHMT2 and methylenetetrahydrofolate dehydrogenase 2 (MTHFD2) — the first and second enzymes in mitochondrial serine catabolism — as independent prognostic markers and potential therapeutic targets across multiple cancer types (40–45).

These multifaceted functions underscore the broad physiological significance of serine catabolism in energy provision, redox equilibrium, and lipid biosynthesis, with its dysregulation being critically implicated in various metabolic disorders and malignancies.

## 3 Serine metabolism and tumors

Cellular metabolic reprogramming is a common feature of human tumors. In conditions of nutrient limitation and stress, tumor cells readjust metabolic pathways to produce sufficient metabolites for their rapid proliferation (46, 47). As one of the most fundamental components of cell structure, amino acids largely support protein synthesis needed for cell proliferation (48). During tumor development, changes in the rate of amino acid uptake, metabolic pathways, metabolites, or key enzymes are referred to as amino acid metabolic reprogramming (49). Serine metabolism also alters during tumor progression, mainly through changes in metabolites and key enzymes (50).

### 3.1 Genes related to serine metabolism

Key enzymes for serine synthesis, such as phosphoglycerate dehydrogenase (PHGDH) and phosphoserine aminotransferase (PSAT), are often abnormally expressed in tumor cells. Oncogenes and tumor suppressor genes also regulate the genes encoding enzymes involved in serine synthesis. The following section primarily introduces the related PHGDH and p53 genes.

#### 3.1.1 PHGDH

PHGDH plays a crucial role in the *de novo* synthesis pathway of serine, acting as both the first enzyme and the rate-limiting enzyme in the reaction process. It is one of the few known metabolic enzymes that are dysregulated in cancer (51–53). Recent studies indicate that activating the serine synthesis pathway (SSP) and upregulating PHGDH expression can promote the growth of various cancer subtypes. Cancer cells use PHGDH and NAD+ to oxidize 10% of the 3-phosphoglycerate (3-PG) produced by glycolysis into the serine precursor 3-phosphohydroxypyruvate (3-PPyr) (54, 55). In addition to catalyzing the first step in serine synthesis, PHGDH accelerates the NADH-dependent reduction of alpha-ketoglutarate (α-KG) to the oncometabolite D-2-hydroxyglutarate (D-2HG), playing an important role in tumorigenesis (56). Amplification of the PHGDH gene on chromosome 1p12 occurs in 6% and 40% of breast cancers and melanomas, respectively (57, 58). In estrogen receptor-negative breast tumors, PHGDH protein levels can be elevated by as much as 70% (7). Increased PHGDH expression levels are also observed in other types of cancers. These studies highlight the important role of PHGDH in tumor biology, offering new directions for further research and cancer treatment.

#### 3.1.2 p53

The tumor suppressor gene TP53 (aka, p53) encodes the protein p53, a crucial regulator of cellular metabolism. p53 is pivotal in the stress response to challenges such as DNA damage, hypoxia, and oncogene activation (59). The complexity and diversity of these cellular processes suggest that, beyond its traditional role as a tumor suppressor, p53 also maintains homeostasis in both normal and cancer cells (60). p53’s capacity to respond to nutritional deficiencies arises from its role as a mediator of cellular stress responses. It is closely related to the ability of cancer cells to cope with serine starvation and oxidative stress. Indeed, p53 is crucial in enabling cancer cells to overcome serine starvation and sustain their antioxidant capacity. Cells lacking p53 exhibit reduced survival and severely impaired proliferative capacity due to a failure to effectively cope with serine starvation and oxidative stress. During serine starvation, the p53-p21 signaling pathway is activated, leading to cell cycle arrest. This arrest reallocates depleted serine for glutathione synthesis, thereby promoting cell survival (9).

#### 3.1.3 PSAT1

PSAT1 (phosphoserine aminotransferase 1), a member of the class V pyridoxal 5’-phosphate-dependent aminotransferase family, is a pivotal enzyme in the three-step serine synthesis pathway (SSP). It catalyzes the transamination of 3-phosphohydroxypyruvate (3-PHP) to phosphoserine using glutamate-derived nitrogen, concomitantly generating α-ketoglutarate (α-KG) (61). PSAT1 overexpression is documented across multiple malignancies — including non-small cell lung cancer (NSCLC), breast cancer, gastric cancer, colon cancer, and ovarian cancer — where it drives tumorigenesis and malignant progression (62–67). Elevated PSAT1 levels correlate with aggressive phenotypes such as proliferation, migration, invasion, and therapy resistance (62, 68–74). Recent evidence demonstrates that PSAT1 inhibition attenuates the tumorigenic potential and pulmonary metastasis of clear cell renal cell carcinoma (ccRCC) (75), while its prognostic utility is increasingly recognized (76). Mechanistically, PSAT1 mediates the reversible conversion of 3-PHP to L-phosphoserine, with glutamate serving as a substrate. Structural analyses reveal tight interactions between L-phosphoserine’s phosphate group and conserved arginine (Arg42, Arg328) and histidine (His41, His327) residues, suggesting that analogs targeting these residues could yield potent inhibitors (77). These findings underscore the therapeutic imperative to develop PSAT1 antagonists.

#### 3.1.4 PSPH

PSPH (phosphoserine phosphatase), the terminal enzyme in L-serine biosynthesis, executes the irreversible dephosphorylation of phosphoserine to serine downstream of PHGDH and PSAT1 in the SSP (34). In melanoma, PSPH upregulation promotes tumor growth *in vitro* and *in vivo*, whereas its knockdown suppresses proliferation (78). Similarly, elevated PSPH expression in breast (79), liver (80), colorectal (81), NSCLC (82), and cutaneous squamous cell carcinomas (83) correlates with enhanced invasiveness and metastasis. Genomic analyses identify frequent PSPH amplifications and rare mutations in 13% of glioblastomas (84). These oncogenic roles position PSPH as a critical node requiring systematic investigation.

#### 3.1.5 Transcriptional regulator ATF4

Activating transcription factor 4 (ATF4), a basic leucine zipper (bZIP) family member, orchestrates metabolic reprogramming by integrating stress signals (e.g., nutrient deprivation, oxidative/ER stress) (85, 86). ATF4 directly activates SSP enzymes (PHGDH, PSAT1, PSPH) to bolster *de novo* serine synthesis (87). Its pro-tumorigenic effects manifest via serine pathway activation in colorectal and breast cancers, where ATF4 knockdown suppresses PHGDH expression and chemoresistance (88, 89). Paradoxically, chronic ER stress-induced ATF4 hyperactivation may upregulate pro-apoptotic factors like CHOP, suggesting context-dependent tumor-suppressive potential (90). This functional duality — shaped by tumor type, microenvironmental stress, and genetic background — positions ATF4 as an emerging therapeutic target. Preclinical studies confirm that ATF4 inhibition synergizes with conventional therapies (91), though clinical translation awaits further validation.

### 3.2 Serine metabolism and the immunosuppressive microenvironment

The immunosuppressive tumor microenvironment (TME) enables immune evasion, with serine metabolism emerging as a key modulator via metabolite allocation, immune cell regulation, and redox control. Serine fuels CD8+ T cell expansion through one-carbon metabolism — a process decoupled from glucose utilization (28). However, tumor cells hijack serine via SSP activation (PHGDH, PSAT1 (68, 92)) or transporters (SLC1A4 (93)), depleting TME serine. This scarcity impairs CD8+ T cell purine synthesis, blunting clonal expansion and IFN-γ production (28). Concurrently, SSP-derived α-KG stabilizes Treg immunosuppression by enhancing KDM5A-mediated Foxp3 demethylation (94). Thus, tumors exploit serine metabolism to simultaneously cripple cytotoxic immunity and bolster Treg activity.

Tumors co-opt serine metabolism (via SSP activation and uptake) to deplete TME serine, directly impairing CD8+ T cells while epigenetically amplifying Treg suppression through α-KG. Targeting this axis — via PHGDH inhibitors or dietary modulation — combined with immune checkpoint blockade may reverse metabolic immunosuppression, offering novel combinatorial therapeutic avenues.

### 3.3 Tumor treatment strategies targeting serine metabolism

#### 3.3.1 Low-serine diet

Serine starvation can induce cellular stress responses and metabolic reprogramming, thereby inhibiting cancer progression (8–10). Studies show that restricting dietary serine and glycine reduces tumor growth in xenograft and allograft models (9, 95). In genetically engineered mouse models of colorectal cancer (driven by inactivation of the tumor suppressor gene Apc) or lymphoma (driven by activation of the oncogene Myc), restricting dietary serine and glycine intake further improved survival by antagonizing the antioxidant response. Disruption of mitochondrial oxidative phosphorylation (using biguanides) results in complex responses that can either enhance or hinder the antitumor effects of serine and glycine starvation. Mouse models of pancreatic and colorectal cancers driven by the Kras oncogene exhibit a weaker response to serine and glycine depletion, reflecting Kras activation, which upregulates enzymes in the serine synthesis pathway and promotes *de novo* serine synthesis (10). Initial analyses of cancer cell lines found a close correlation between glycine consumption from the biosynthetic pathway and cell proliferation rates (22). However, subsequent studies revealed that serine, not glycine, is the amino acid consumed most rapidly for proliferation, with some cells resorting to glycine only when serine is depleted (8). Recent studies also show that inhibiting PHGDH enhances the therapeutic effects of a serine-deficient diet (96). Recent *in vivo* studies have demonstrated that combined dietary intervention and pharmacological inhibition exhibit significant therapeutic efficacy against tumors resistant to single-modality treatments, with evidence of reduced one-carbon metabolic flux (17). Concurrently, our findings reveal that serine deprivation in cancer cells induces mitochondrial dysfunction, leading to cytoplasmic accumulation of mitochondrial DNA (mtDNA) and activation of the cGAS-STING signaling pathway, thereby stimulating tumor-targeted immune responses. This effect is synergistically enhanced when combined with PD-1-targeted therapy (97).

Notably, the role of serine in cancer therapy exhibits duality: studies indicate that serine critically regulates estrogen receptor α (ESR1) gene expression by maintaining histone acetylation levels, and ESR1 expression directly determines the sensitivity of hormone receptor-positive breast cancers to endocrine therapies such as tamoxifen (98). Paradoxically, downstream metabolites of serine catabolism, including formate, exert beneficial effects on antitumor T cells. Formate supplementation synergizes with PD-1 blockade (99). Importantly, serine-restricted diets alone may inadvertently stimulate resistance mechanisms such as PD-L1 upregulation, underscoring the necessity to combine dietary interventions with PD-L1-targeted strategies (100).

#### 3.3.2 PHGDH inhibitors

Research into PHGDH’s role in regulating cell growth has deepened, establishing it as a promising direction for targeted drug discovery (92). In 2018, Ishida et al. demonstrated that interfering with the SOG (serine synthesis pathway) using PHGDH inhibitors can induce synergistic cell death *in vitro* and *in vivo* (101). Studies show that CBR-5884, a PHGDH inhibitor, has dual effects: it inhibits cell proliferation and increases sensitivity to hypoxia-induced cell death (102). Recent studies indicate that PHGDH expression is reduced by 40-50% in 1p/19q-codeleted gliomas compared to non-codeleted gliomas, suggesting these gliomas have selective vulnerability to serine and glutathione depletion (103).

Numerous PHGDH inhibitors have been reported and can be classified into two categories based on their binding sites: allosteric inhibitors and orthosteric inhibitors. Allosteric inhibitors include the CBR-5884 series, bis-sulfonamide derivatives, piperazine-1-thiocarboxamide scaffolds, α-thio-oxamidamide scaffolds, and pyrazole-5-thiocarboxamide scaffolds, among others. Orthosteric inhibitors include indoleamide derivatives, phenyl-pyrazole-5-carboxamide derivatives, and certain fragments. The characteristics of these inhibitors are summarized in **Table 2**.

**Table 2:** Types and action characteristics of PHGDH inhibitors.

Inhibitor	Variety	Feature
Allosteric inhibitors Allosteric inhibitors	CBR-5884	CBR-5884 inhibited PHGDH substrates (3-PG and NAD-TUTA) in a non-competitive and time-dependent manner, but it was not stable in mouse plasma, and its chemical structure still needed to be optimized.
	Disulfram derivatives	Dithioethers inhibit PHGDH by oxidizing the Cys116 residue, inducing the active tetramer to an inactive dimer or a less active monomeric intermediate.
	Piperazine-1-carbothioamide scaffold	NCT-503 showed selective activity against PHGDH-dependent cell lines and xenograft tumors by affecting the oligomerization state of PHGDH.
	ɑ-Ketothioamide scaffold	In a - ketone thiourea for all three parts (a, B, C) after a thorough research, there are only two compounds, 28 generation of carbonyl compounds with sulfur and 29 with sulfonyl hydrazide compounds showed moderate enzyme activity. Cells (CETSA) show that the thermal drift analysis, 28 and 29 can stable PHGDH protein, further confirmed they interact with cells PHGDH in the pyrolysis products.
	PKUMDL-WQ series	PKUMDL – and PKUMDLWQ WQ - 2101-2201 *in vivo* selectively inhibit PHGDH amplification MDA - MB - 468 transplanted tumor, without affecting the MDA - MB231 transplantation tumor growth.
	Natural products as PHGDH inhibitors	Nitrogen zhuo methadone E (54) was first isolated from natural products (aspergillus flavus) get PHGDH inhibitor, another kind of natural products, nuts lactone A (Lox A, 55) separated from the feed. The natural bitter almond lactone has obvious PHGDH inhibitory activity.
Orthosteric inhibitors	Phenylpyrazole-5-carboxamide derivatives	Pka of acid on the inhibitory activity plays an important role, because sulfonyl inhibitory activity was greatly enhanced by the introduction of the acetate.
	Fragment hits for PHGDH	Since these promising fragments, can use drug design based on fragment to explore more effective competitive PHGDH nadrode inhibitors

#### 3.3.3 Other inhibitors

Serine hydroxymethyltransferase (SHMT), a pivotal enzyme in one-carbon metabolism, catalyzes the conversion of serine and tetrahydrofolate (THF) to glycine and 5,10-methylenetetrahydrofolate (5,10-CH_2_-THF), providing one-carbon units for nucleotide synthesis. SHMT is overexpressed in various cancers and strongly associated with tumor proliferation and chemoresistance (22). Recent advances in targeting SHMT have positioned it as a hotspot for metabolic therapy. Pemetrexed, an antifolate chemotherapeutic agent used in non-small cell lung cancer and breast cancer, competitively inhibits cytosolic SHMT1 activity by forming hydrogen bonds at the active site, mimicking THF binding. However, pemetrexed exhibits polypharmacology by targeting multiple one-carbon metabolic enzymes (104). SHIN1, a dual small-molecule inhibitor of SHMT1/2, achieves suppression via competitive THF binding and hydrogen bond interactions between its –NH group and SHMT1/2 (105). Notably, the antidepressant sertraline has recently been identified as a competitive dual SHMT1/2 inhibitor through direct binding to both isoforms (106).

MTHFD2, whose expression is upregulated in tumors such as breast and colorectal cancers, correlates with poor survival (44, 107). DS18561882, a potent and orally bioavailable MTHFD2 inhibitor with demonstrated *in vivo* antitumor efficacy, has emerged as a promising candidate for breast cancer treatment. However, its biological roles in other malignancies remain to be elucidated (108).

### 3.4 Traditional Chinese medicine affects serine metabolism

The biosynthesis of serine and glycine is crucial in tumorigenesis (54). Research indicates that a deficiency in serine and glycine reduces glutathione synthesis and increases reactive oxygen species (ROS) levels in cells (32). Moreover, downregulating the rate-limiting enzymes of serine/glycine metabolism, such as phosphoserine phosphatase (PSPH) and phosphoserine aminotransferase 1 (PSAT1), may disrupt the pro-tumor effects mediated by serine/glycine metabolism (7, 75). Targeting amino acid metabolism shows promise for discovering selective inhibitors and new strategies for cancer treatment (109). Recent studies suggest that certain traditional Chinese medicine (TCM) herbs and compounds may exhibit antitumor effects by targeting amino acid metabolic pathways, highlighting TCM’s potential in cancer therapy. Licorice is a typical TCM herb commonly used to treat inflammation and allergies. Recent research suggests that licorice root extract exhibits anticancer effects in nasopharyngeal carcinoma cells. Metabolomic analysis revealed that these anticancer effects may relate to the downregulation of fatty acid biosynthesis metabolites and reductions in glutamic acid, serine, and threonine metabolism (110). Tanshinones, bioactive constituents derived from Salvia miltiorrhiza, have recently been demonstrated to upregulate PHGDH mRNA expression levels (111). Unfortunately, research on how TCM compounds or decoctions regulate serine metabolism and their effects on cancer cell invasion and migration remains scarce. The mechanisms remain unclear and warrant further in-depth studies.

## 4 Summary and outlook

Serine metabolism is crucial in tumor development, and tumor-related biomarkers of this metabolism show promise for clinical applications. In the serine synthesis pathway, key enzymes like PHGDH and PSPH are upregulated in specific tumor types (51, 52, 112). Abnormal expression of PHGDH and PSPH may enhance serine synthesis in tumor cells, positioning them as potential diagnostic biomarkers. Serine metabolic enzymes have also gained attention in tumor research. SHMT2, one of the key enzymes in serine metabolism, is upregulated in certain tumors (113–117). Serine aminotransferase 1 (SAT1) is closely linked to serine metabolism, with abnormal expression associated with tumor development (118, 119). The abnormal expression of these enzymes may affect the metabolic pathways of tumor cells, further influencing tumor proliferation and survival. Thus, they may serve as potential markers for tumor diagnosis and treatment. Research on serine and its metabolites has significant application potential. Alterations in glycine and cysteine concentrations may be linked to tumor development and metabolic disorders (120, 121). In various cancers, serine metabolism, one-carbon (1C) metabolism, and mTOR signaling pathways show abnormal hyperactivation (32, 122, 123). Serine and one-carbon metabolism may serve as key links between mTOR signaling and DNA methylation, promoting tumor growth (124). It is noteworthy that although PHGDH is often amplified in various types of cancer, therapeutic interventions targeting PHGDH are far more complex than initially imagined. This complexity stems from the fact that PHGDH has both tumor-promoting and potential anti-metastatic effects (125, 126). Additionally, exogenous serine can sometimes compensate for the deficiencies caused by the loss of serine biosynthesis (127–129). These findings imply that simply inhibiting PHGDH may not suffice for effective tumor treatment due to its complex dual role in tumor development. Thus, developing effective therapeutic strategies targeting PHGDH necessitates a deeper understanding of its specific functions and regulatory mechanisms in tumors, along with its effects on normal cells. Furthermore, supplementing exogenous serine may pose challenges to therapeutic interventions. Although serine supply can be externally supplemented, achieving a balance in the serine metabolic pathway remains complex for tumor cells. Therefore, relying solely on exogenous serine as a treatment strategy may be infeasible, necessitating comprehensive consideration of the overall regulation of the serine metabolic pathway. Combining these biomarkers with other clinical indicators could enhance the efficacy of tumor diagnosis and analysis. Investigating the link between serine metabolism and tumor development, identifying additional biomarkers, and enhancing research on serine metabolism and related pathways will deepen our understanding of tumor mechanisms and potentially yield new targets and strategies for diagnosis and treatment.
